# Community involvement in a juvenile partner justice behavioral health

**DOI:** 10.1186/1940-0640-10-S1-A34

**Published:** 2015-02-20

**Authors:** Carl Leukefeld, Danica Knight, Steven Belenko, Angela Robertson, Tisha Wiley, Gail Wasserman, Ralph DiClemente, Gene Brody, Hannah Knudsen

**Affiliations:** 1 College of Medicine, University of Kentucky, Lexington, KY, 40506, USA; 2 Institute of Behavioral Research, Texas Christian University, Fort Worth, TX, 76129, USA; 3 Department of Criminal Justice, Temple University, Philadelphia, PA, 19122, USA; 4 Mississippi State University, Starkville, MS, 39762, USA; 5 Services Research Branch, National Institute on Drug Abuse, Rockville, MD, 20852, USA; 6 Columbia University, New York, NY, 10027, USA; 7 Emory University, Atlanta, GA, 30322, USA; 8 Institute for Behavioral Research, University of Georgia, Athens, GA, 30602, USA

## Service organizational implementation trial

The U.S. juvenile justice system has a number of possible behavioral health service community linkages for substance abuse and HIV services. However, there have only been a small number of systemic studies that examine and seek to improve these community behavioral health linkages for involved youth. Implementation science, as an emerging area, is a way of identifying, testing, and understanding effective strategies for translating treatment and prevention evidence-based approaches into community behavioral health service delivery. The purpose of this presentation, within the context of the Juvenile Justice-Translating Research Interventions for Adolescents in the Legal System (JJ-TRIALS) implementation behavioral health trial, is to describe the diverse settings in which the study will be initiated and how partners are involved in the study design. State partners include juvenile justice (JJ) and behavioral health services organizations in seven states (Florida, Georgia, Kentucky, Mississippi, New York, Pennsylvania, Texas) and the District of Columbia. Factors associated with selection, participation, and implementing the study in 36 sites will be explored. Each site will include a JJ agency and up to two community behavioral health service organizations for 7–10 implementation team members representing executive, management, supervisory, and line staff. JJ partner involvement is helping to design the study protocol that is both feasible for sites and meets partner needs. Study sites are defined as a county or service area, which can include multiple counties when behavioral health services are sparse. Site clusters will be used, which are defined within each state to assure that each of the six research centers have an equal number of experimental and control sites to take into account broader contextual factors. Options for defining clusters include county size, JJ youth population, urban/rural, or type of community supervision — probation or juvenile drug court. Sites will not be preselected for substance use or related behavioral health service needs, so youth are included with diverse behavioral health needs. Two JJ partners have actively participated on the study design work group, and the protocol has been vetted through partners at steering committee meetings and conference calls.

